# Two new genera and species of the termite symbiont lineage Termitohospitini (Coleoptera, Staphylinidae, Aleocharinae) from Bolivia and peninsular Malaysia

**DOI:** 10.3897/zookeys.254.4043

**Published:** 2012-12-21

**Authors:** Taisuke Kanao, K. Taro Eldredge, Munetoshi Maruyama

**Affiliations:** 1 Entomological Laboratory, Graduate School of Bioresource and Bioenvironmental Sciences, Kyushu University, Fukuoka, 812-8581 Japan; 2 Department of Ecology and Evolutionary Biology, and Division of Entomology Biodiversity Institute, 1501 Crestline Dr., Suite 140, University of Kansas, Lawrence, KS 66045-2811, U. S. A.; 3 The Kyushu University Museum, Fukuoka, 812-8581 Japan

**Keywords:** *Coptotermes*, Hetairotermitina, Termitohospitina, limuloid, Masuriini, Myllaenini, physogastry, termitophily

## Abstract

*Coptotermocola clavicornis* **gen. & sp. n.** and *Neotermitosocius bolivianus* **gen. & sp. n.** of the termite inquilinous tribe Termitohospitini are described from peninsular Malaysia and Bolivia, respectively. The Termitohospitini are most readily diagnosable by the distally migrated anterior tentorial pits that are no longer associated with the antennal fossae, and by the enlarged vertex which obscures the antennal fossae dorsally. Additionally, the Termitohospitini are hypothesized to share a recent common ancestor with the Masuriini and Myllaenini due to shared derived morphologies of the lacinia distal teeth with lateral cuticular processes, presence of a unique maxillary palpomere III sensilla, and anterolateral angles of mentum produced. Habitus photographs and illustrations of diagnostic features are provided for the two new genera in order to facilitate future work.

## Introduction

Eusocial insect colonies are often accompanied by a unique faunal assembly that takes advantage of available colony resources (e.g. Hölldobler and Wilson 1990, Wheeler 1928). Symbionts of termites are commonly referred to as termitophiles (Kistner 1969). A large number of termitophiles have evolved within the beetle family Staphylinidae, particularly in lineages of the subfamily Aleocharinae. Among the recognized 58 tribes (Bouchard et al. 2011), 1,151 genera and 12,851 species (Thayer 2005) of Aleocharinae, approximately 650 species and 190 genera among 17 tribes have recorded associations with termites, of which eleven tribes are comprised exclusively of termitophilous species (Kanao unpublished data).

Termitophilous Aleocharinae that exhibit relatively complex symbiosis with their host termites have repeatedly converged on physogastric and limuloid body plans (e.g. Figs 24–27). Physogastry is the result of abdominal or thoracic inflation and is hypothesized to serve a mimetic function (Kistner 1969). Physogastric species are generally well integrated into the termite social system, actively interacting with their hosts and slow moving. On the other hand, limuloid taxa are teardrop-shaped and the body plan is hypothesized to serve a defensive function (Kistner 1979). Limuloid taxa often also exhibit extremely compact appendages and cavities to receive structures more vulnerable to host aggression (e.g. distal podomeres). In contrast to physogastric species, limuloid species are faster moving and usually do not actively interact with their hosts.

Among the aleocharine termitophiles, the tribe Termitohospitini Seevers, 1941 is comprised exclusively of termite symbiotic species. This tribe was originally proposed by Seevers (1941) to separate a handful of Neotropical termitophilous species of the tribe Bolitocharini (presently Homalotini Heer, 1839). Seevers (1957) later expanded Termitohospitini to include termitophiles from other biogeographic regions based on the 4-4-5 tarsal formula and proposed four subtribes: Termitohospitina Seevers, 1941 for Neotropical members; Hetairotermitina Seevers, 1957 for Australian and Oriental members; Termitospectrina Seevers, 1957 for Pantropical members; Termitusina Seevers, 1957 for Ethiopian members. Kistner and Jacobson (1976) subsequently raised Termitusina to tribe with Termitospectrina as a subtribe within it, based on 4-articled maxillary and 3-articled labial palpi. Therefore, Termitohospitini is presently divided into two subtribes, Termitohospitina and Hetairotermitina. Termitohospitina includes 15 species distributed among five genera (Mann 1923, Silvestri 1947, Seevers 1957, Borgmeier 1959) and Hetairotermitina includes 21 species distributed among seven genera (Lea 1910, Cameron 1920, 1950, Seevers 1957, Kistner 1970, 1976, 1985, Abdel-Galil and Kistner 1987, Roisin and Pasteels 1990, Oke 1933, Pace 1993, Maruyama and Iwata 2002).

Most species of Termitohospitini ancestrally share a limuloid body form but a few taxa have secondarily evolved physogastric forms (Kanao et al. in preparation). Notably, the species of *Coptoxenus* Kistner, 1976 exhibits physogastry of the thorax, a peculiar condition among aleocharines. With a diversity of body forms, the tribe offers a unique opportunity to study the independent evolution of adaptive morphologies as it correlates with integration and behavior with host termite societies. However, as with most termitophiles, basic understanding of Termitohospitini biodiversity lags behind that of their free-living relatives. Inquilinous groups are difficult to collect and relatively few specimens are available for comprehensive understanding of their biodiversity. Here we describe two new genera and species of Termitohospitini as part of our ongoing research on the evolution of termitophily in termitohospitines. We provide habitus photographs, illustrations of diagnostic features, a new tribal diagnosis as an update to previous diagnoses (Seevers 1941, 1957), and putative synapomorphies that support a close relationship of Termitohospitini with Masuriini and Myllaenini.

## Materials and methods

The first author conducted fieldwork at Ulu Gombak, which is approximately 25 km northwest of Kuala Lumpur, Malaysia in 2010, 2011 and 2012. Specimens of a new genus collected at the site for this study were initially preserved in 2.0 ml vials of 80% ethanol. The other undescribed genus was discovered in the Snow Entomological Museum Collection at the University of Kansas.

The technical procedures used for this study generally follow those described in Maruyama (2006). When a permanent mount was made, cleared specimens or parts were dehydrated by successively transferring specimens to higher ethanol (EtOH) concentrations until finally reaching 100% EtOH, and then placed into Euparal. When appropriate, dissections were made within Euparal on a glass mounting substrate. Full body dissections were made and preserved on glass microscope slides. Dissected body parts were preserved and mounted on halved glass cover slips, subsequently glued onto a halved paper glue board, and mounted under the respective specimen (see Maruyama 2004, for details).

In the descriptions, macrochaetotaxy formulas depict raw numbers of setae and not the numbers of setal pairs. Additionally, the number of macrosetae on tergite IX and X refers to one side of the body and therefore the numbers of setal pairs.

**Table 1.** Checklist of described species of Termitohospitini. Asterisks indicate type species.

**Tribe Termitohospitini**
**Subtribe Hetairotermitina**
***Coptophysa* Roisin & Pasteels, 1990**
*Coptophysa obesa* Roisin & Pasteels, 1990
***Coptophysella* Roisin & Pasteels, 1990**
*Coptophysella pulposa* Roisin & Pasteels, 1990
***Coptotermocola* gen. n.**
*Coptotermocola clavicornis* **sp. n.**
***Coptoxenus* Kistner, 1976**
*Coptoxenus thronileyi* Kistner, 1976
***Hetairotermes* Cameron, 1920**
*Hetairotermes agilis* Cameron, 1920
*Hetairotermes borealis* Kistner, 1970
*Hetairotermes bryanti* Cameron, 1950
*Hetairotermes capitalis* Kistner, 1970
*Hetairotermes gayi* Kistner, 1970
*Hetairotermes greavesi* Kistner, 1970
*Hetairotermes barretti* Cameron, 1950
*Hetairotermes hirsutus* Kistner, 1970
*Hetairotermes insulanus* Seevers, 1957
*Hetairotermes latevricola* (Lea, 1910) *
*Hetairotermes occidentalis* Kistner, 1970
*Hetairotermes piceus* Cameron, 1920
*Hetairotermes punctiventris* (Lea, 1910)
***Japanophilus* Maruyama & Iwata, 2002**
*Japanophilus hojoi* Maruyama & Iwata, 2002
***Sinophilus* Kistner, 1985**
*Sinophilus rougemonti* Pace, 1993
*Sinophilus xiai* Kistner, 1985 *
*Sinophilus yukoae* Maruyama & Iwata, 2002
***Termitobra* Seevers, 1957**
*Termitobra perinthoides* Seevers, 1957
**Subtribe Termitohospitina**
***Blapticoxenus* Mann, 1923**
*Blapticoxenus brunneus* Mann, 1923
***Noetermitosocius* gen. n.**
*Noetermitosocius bolivianus* **sp. n.**
***Paratermitosocius* Seevers, 1941**
*Paratermitosocius vestitus* (Mann, 1923)
***Termitohospes* Seevers, 1941**
*Termitohospes brasiliana* Seevers, 1957
*Termitohospes guianae* Seevers, 1941
*Termitohospes limulus* Seevers, 1957
*Termitohospes miricroniger* Seevers, 1941 *
*Termitohospes nitens* Borgmeier, 1959
*Termitohospes panamensis* Seevers, 1941
*Termitohospes punctulatus* Borgmeier, 1959
*Termitohospes silvestrii* Seevers, 1957
*Termitohospes tachyporoides* Seevers, 1957
*Termitohospes unicolor* (Silvestri, 1947)
***Termitosodails* Seevers, 1941**
*Termitosodails barticae* Seevers, 1941 *
*Termitosodails fasciatus* (Silvestri, 1947)
***Termitosocius* Seevers, 1941**
*Termitosocius microps* Seevers, 1941

## Taxonomy

### Termitohospitini

Seevers, 1941

#### Type genus:

*Termitohospes* Seevers, 1941.

#### Diagnosis.

Members of the tribe Termitohospitini most closely resemble the tribe Myllaenini and Masuriini (*sensu* Ahn and Ashe 2004), and are hypothesized to be closely related based on the following combination of putative synapomorphies: 1) subapical marginal setae of maxillary lacinia with basal, paired cuticular processes (spinose scales of Ahn and Ashe 2004); 2) presence of a unique sensory patch on lateral surface of maxillary palpomere III; 3) anterolateral angles of mentum produced.

Species of Termitohospitini are diagnosable from all other Aleocharinae, including Myllaenini and Masuriini, by the following putative synapomorphies: 1) anterior tentorial arms of the head migrated anteriorly and disassociated from antennal fossae (Seevers 1941); 2) antennal fossae dorsally obscured by enlarged vertex (Seevers 1941); 3) maxillary lacinia with basal paired cuticular processes reduced in size; 4) processes of anterolateral angles of mentum reduced in size; 5) ligula broad and reduced in length.

### Neotermitosocius

Kanao, Eldredge & Maruyama gen. n.

urn:lsid:zoobank.org:act:7CEBD279-F24B-4BA5-A2F6-2496848C786E

http://species-id.net/wiki/Neotermitosocius

#### Type species:

*Neotermitosocius bolivianus* sp. n.

#### Diagnosis.

**Figures 1–2.**
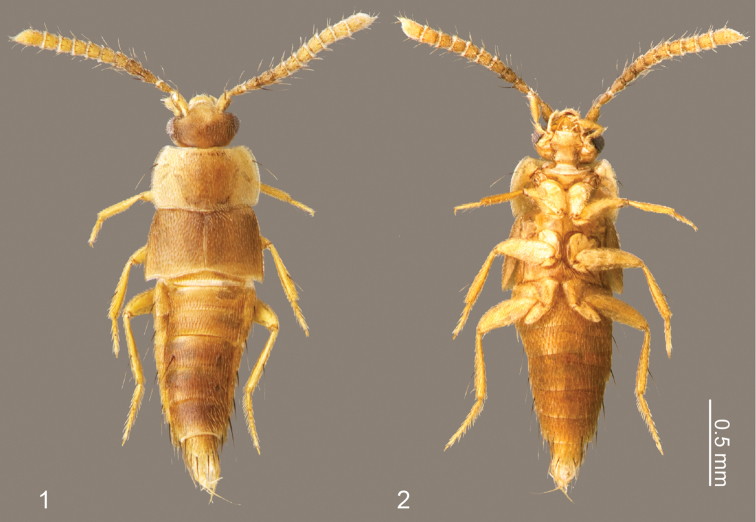
Habitus of *Neotermitosocius bolivianus***.** **1** dorsal view **2** ventral view.

This monotypic genus is distinguishable from other Termitohospitini by its slender parallel-sided body, longer legs and apically truncate pronotum, which dorsally exposes much of the head (Fig. 1).

#### Description.

Overall shape (Fig. 1) parallel-sided and somewhat dorsoventrally flattened. Posterior one-fifth of head covered by pronotum (Fig. 1).

**Figures 3–4. F2:**
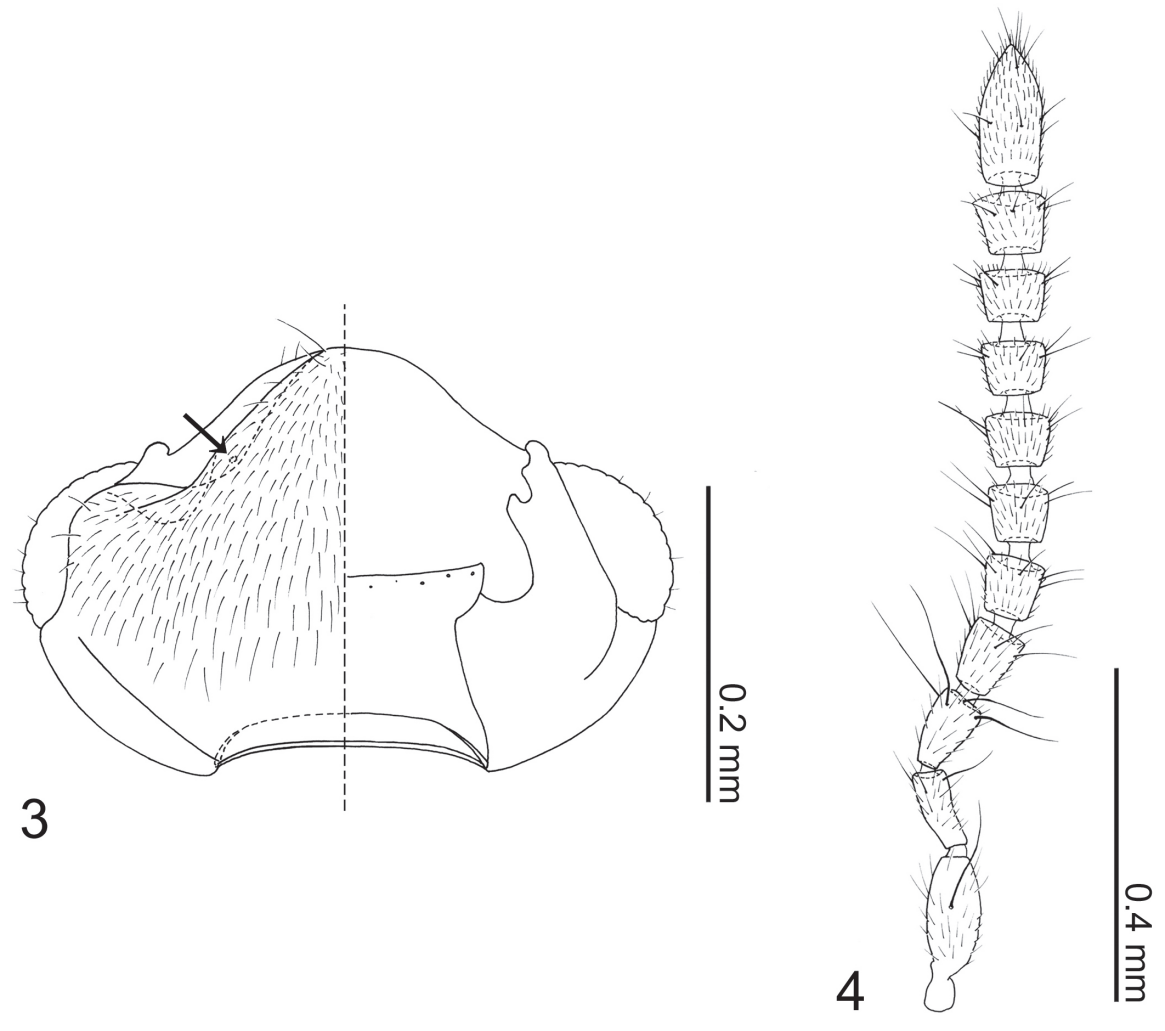
*Neotermitosocius bolivianus*. **3** head capsule, left side = dorsal view, right side = ventral view **4** antenna.

**Figures 5–10. F3:**
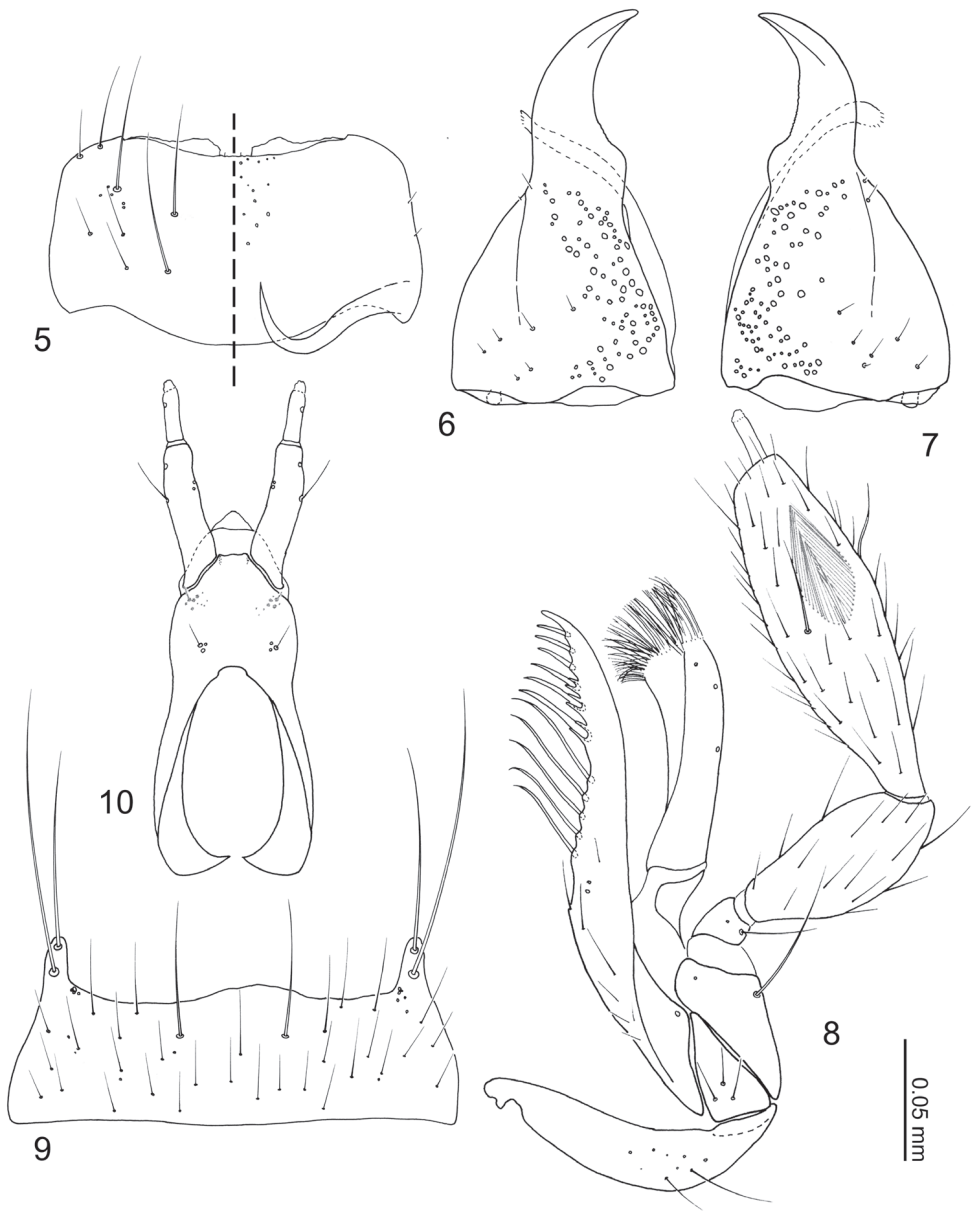
*Neotermitosocius bolivianus* mouthparts. **5** labrum, left side = labrum, right side = epipharynx **6** left mandible, dorsal view **7** right mandible, dorsal view **8** maxilla, ventral view **9** mentum, ventral view **10** labium in ventral view.

Head (Fig. 3) transverse, widest at eyes. Antennal fossae deep, partially dorsally obscured by vertex. Eyes large and produced anterolaterally. Antennae (Fig. 4) with 11 articles; antennal articles compact and article pedicles scarcely visible externally; antennomeres I and XI longest; antennomere I partially obscured by enlarged antennal fossae; antennomere II approximately three times longer than wide, dilated at apex, narrower than other articles; antennomere III trapezoidal, apex twice as wide as base; antennomere IV trapezoidal, slightly shorter than antennomere III; antennomere V–X successively widening distally; antennomere XI narrowed apically. Labrum (Fig. 5) transverse, with pores at middle, anterior margin of membranous area concave; epipharynx (Fig. 5) relatively smooth, midline with pores from apex to middle. Mandibles (Figs 6–7) almost symmetrical, apex acute, dorsally covered with numerous pores, with anterior one-fifth curved adorally, moderately developed tooth present at middle of adoral margin, base of scrobe strongly laterally developed. Right mandible (Fig. 7) apex slightly more acute. Maxillary (Fig. 8) lacinia elongate, adorally with 11 strong setae; apical three setae shortest, preceding basal three setae with cuticular processes (spinose scale of Ahn and Ashe 2004) on either side of base; galea as long as lacinia, slightly dilated apically, apex densely setulate; palpus with 4 articles; article I trapezoidal; article II oval, narrowed at base; article III approximately twice as long as article II, more than three times longer than wide, widest at middle, sparsely covered with long setae, sensory patch near middle of lateral surface; article IV narrow and parallel-sided, approximately one-fourth as wide as apical margin of article III; pseudosegment poorly delimited and inconspicuous. Mentum (Fig. 9) trapezoidal, sparsely covered with setae and small pseudopores; anterior margin bisinuate, with a pair of long setae at middle; anterolateral corners produced and with 2 long setae; posterior margin almost straight. Labial palpus (Fig. 10) with 3 articles, first article a fusion of I + II; article I + II with a seta (probably homologous with seta h of Sawada [1972]) at outer medial margin, twin and medial pores present; article III half as long as article I, with a pore at anterolateral corner; ligula triangular with posterior half sclerotized; prementum wider than long, disc with a pair of setal pores and two pair of real pores present; apodemes broad and longer than disc, dilated posteriorly with apices recurved internally and almost touching.

**Figures 11–16. F4:**
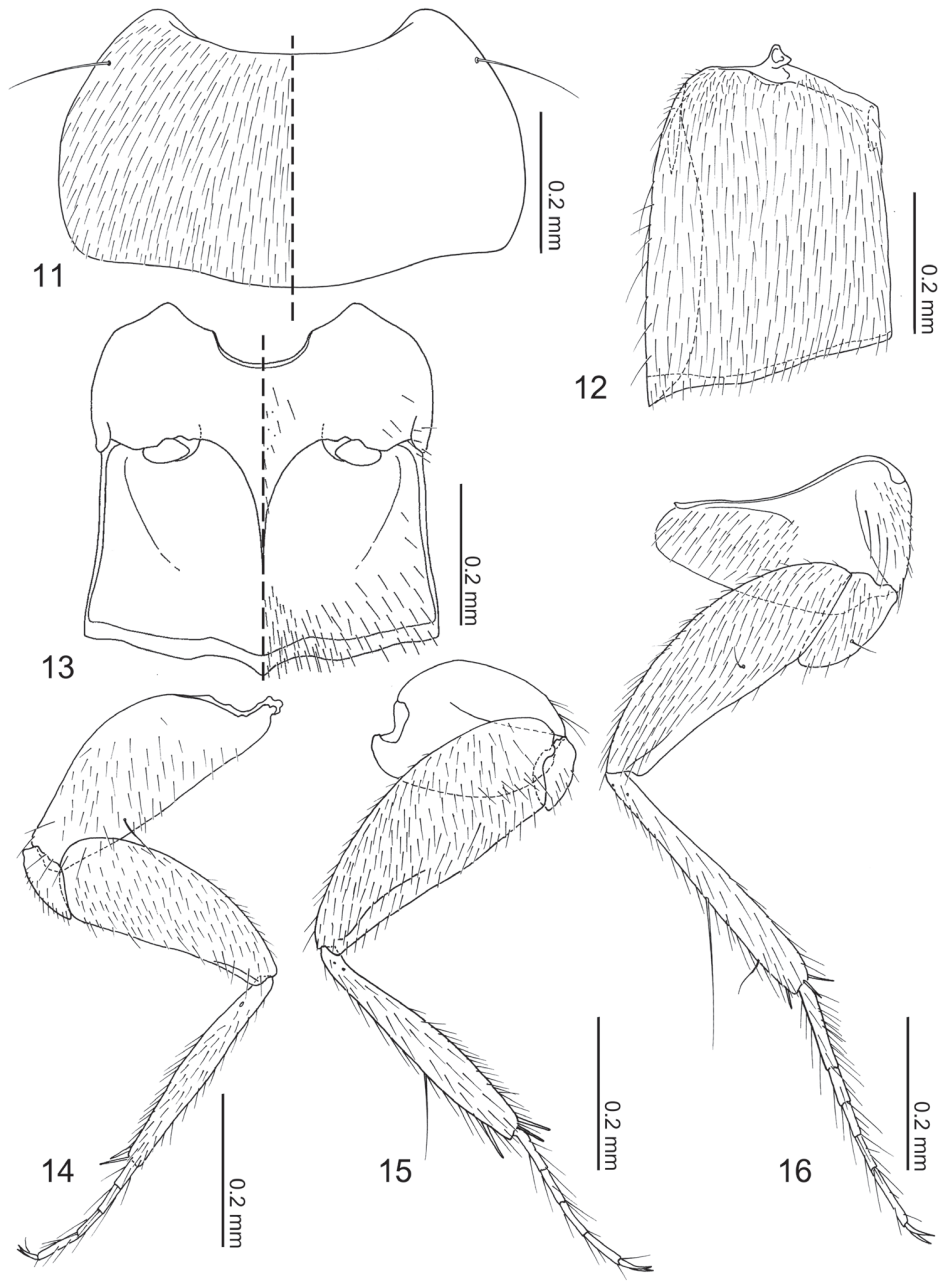
*Neotermitosocius bolivianus*, thorax and legs. **11** pronotum, left side with setae **12** elytron, left **13** meso- and metaventrites, anatomical left side with setae **14** fore leg, posterior surface **15** mid leg, anterior surface **16** hind leg, anterior surface.

Pronotum (Fig. 11) transverse, widest near middle; anterior margin broadly concave; posterior margin slightly rounded. Prosternum reduced in length. Elytra (Fig. 12) subquadrate, longer than wide with lateral margin deflexed. Wings fully developed. Mesoventrite (Fig. 13) short, approximately half as long as metaventrite; mesoventral process developed posteriorly and carinate; mesocoxal cavity narrowly separated and marginal bead complete. Legs (Figs 14–16) overall slender; tarsal formula 4-4-5; tarsomeres slender and parallel-sided. Fore leg (Fig. 14) with coxa subequal in length to femur; trochanter subtriangular; femur narrowed apically; tibia thin; tarsomeres I–III subequal in length, tarsomere IV longer. Mid leg (Fig. 15) with coxa oval; trochanter small; femur narrowed apically; tibia thin, slightly dilated subapically, one macroseta present on dorsal surface; tarsomeres subequal in length. Hind leg with coxa (Fig. 16) subtriangular; trochanter subtriangular, macroseta present on anterior surface; femur slightly narrowed apically; tibia thin, slightly dilated apically; tarsomere I longer than II + III combined, tarsomere V twice as long as IV.

**Figures 17–23.**
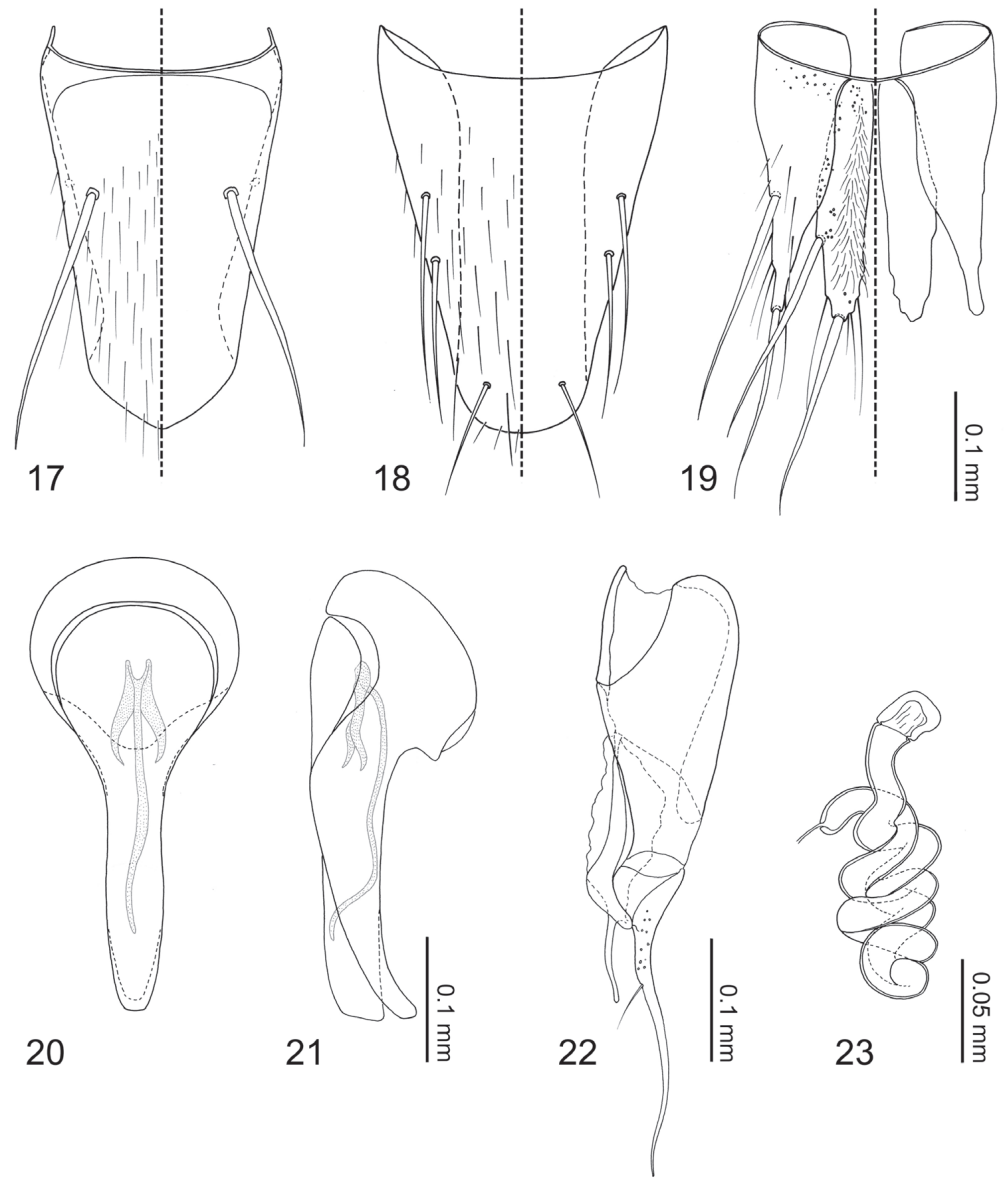
*Neotermitosocius bolivianus*, abdominal sclerites and genitalia. **17** tergite VIII **18** sternite VIII **19** tergites IX–X **20** median lobe of aedeagus, aparameral view **21** median lobe of aedeagus, lateral view **22** paramere, external view **23** spermatheca.

Abdomen (Figs 1–2) narrowed posteriorly. Segment I represented only by tergite I fused to metanotum. Segment II represented only by tergite II. Segments III–VII with 1 tergite, 1 sternite, and 2 pairs of paratergites respectively. Tergite VIII (Fig. 17) narrowed posteriorly, posterior margin slightly medially produced. Sternite VIII (Fig. 18) with posterior margin rounded. Tergite IX (Fig. 19) fully subdivided dorsally by tergite X; tergite X (Fig. 19) fully divided medially to base. Tergite X divided, only connected each other and tergite IX on anterior margin.

Median lobe of aedeagus (Figs 20–21) with basal capsule bulbous, more than three times as wide as apical lobe (Fig. 20), paramerally expanded (Fig. 21). Paramere (Fig. 22) with a structure appearing homologous to Seevers’ (1978) medial phragma produced apically; apical lobe extremely elongate and tapered apically, subequal in length to paramerite, single seta present. Spermatheca (Fig. 23) long and coiled.

#### Etymology.

The generic name is derived from a combination of *Neo*, Greek for “new” and the name of a similar-looking genus *Termitosocius* Seevers, 1941. The gender is masculine.

### Neotermitosocius bolivianus

Kanao, Eldredge & Maruyama sp. n.

urn:lsid:zoobank.org:act:1625FEFE-B75F-4C81-A9F9-65DAA629F1E5

http://species-id.net/wiki/Neotermitosocius_bolivianus

Figures 1 [Fig F2] [Fig F3] [Fig F4] –23

#### Type material.

*Holotype*: ♂, “BOLIVIA: Cochabamba/117 km E Yungas/(Cochabamba-Villa Tunari Rd)/17°6'32"S; 65°41'12"W/ca. 1,040 m alt., 1–6-II-1999; R. Hanley ex. flight/intercept trap BOL1H99 028//SMO754884/KUNHM-ENT”.

*Paratypes*: 2♂♂, ♀, same date and locality data as holotype (♂, SEMC barcode number “SMO75489”, completely disarticulated; ♂, SEMC barcode number “SMO75488”, abdominal segments VII–X dissected off; ♀, SEMC barcode number “SMO754880”, head and abdominal segments VIII–X dissected, spermatheca not recovered). ♂, ♀, ?, same locality data as the holotype, differing data reads “8–10-II-1999”, “BOL1H99 062” (♂, SEMC barcode number “SMO 754864”, abdominal segments VIII–X dissected off; ♀, SEMC barcode number “SMO754858”, completely disarticulated; ♀, SEMC barcode number “SMO754860”, right antennomeres VI–XI and abdominal segments VIII–X dissected off; ♀, SEMC barcode number “SMO754863” and additional label reading “Termitohospitini/new genus/det. K. T. Eldredge 2011”, abdominal segments VIII–X dissected off; ?, SEMC barcode number “SMO754857”, hind wings removed); ♂, “BOLIVIA: Cochabamba/117 km E. Cochabamba, ai/Lagunitas, 1000m, 17°6'22"S/65°40'57"W, 6–8-II-1999/F. Genier, mountain evergreen/forest, ex. flight intercept trap/99-037// SMO754908/KUNHM-ENT”, completely disarticulated.

All type specimens are deposited in the Snow Entomological Museum Collection (SEMC).

#### Diagnosis.

This species is diagnosable based on the generic diagnosis above.

#### Description.

Body (Figs 1–2) approximately 2 mm in length (1.84–2.00 mm, N = 2), almost uniformly orange brown, but head slightly darker. Dorsal surface of head (Fig. 3) sparsely covered with setae, a pair of long setae present at anterior margin of clypeus. Eyes with uniform cover of inter-ommatidial setae. Antennae (Fig. 4) sparsely covered with setae; antennomere I with a long macroseta at middle of internal lateral surface; antennomere II with 4–5 macrosetae around apical margin, one macroseta conspicuously longer; antennomere III with 4 thick, very long macrosetae at apical margin; antennomeres IV–X with 3–4 macrosetae around apical margin; antennomere XI with several macrosetae near middle and apex. Labrum (Fig. 5) dorsal surface with 2 pairs of setae at anterolateral corners, 3 pairs of longer and 3 pairs of shorter setae at mesal area of disc; epipharynx (Fig. 5) with two pairs of lateral setulae. Mandible (Figs 6–7) dorsum with 2 pairs of scrobal setae near middle of aboral margin, six setae around base of disc. Maxillary (Fig. 8) lacinia with 2 pores at middle; galea with 3 pores apicomedially; maxillary palpal article I with a long seta at lateral margin and a medial pore, article II sparsely covered with setae, article III sparsely covered with setae and 2 stronger setae medially.

Pronotum (Fig. 11) transverse (pronotum length = 0.30–0.40 mm, pronotum width = 0.54–0.72 mm, N = 7), densely covered with setae, with a pair of long macrosetae at anterolateral corners. Elytra (Fig. 12) longer than wide (elytra length = 0.42–0.44 mm, elytra width = 0.30–0.34 mm, N = 5), densely covered with setae, setae of lateral margins longer. Mesoventrite (Fig. 13) sparsely covered with minute setae at central and posterolateral areas. Metaventrite (Fig. 13) posterior one third sparsely covered with setae. Fore leg (Fig. 14) uniformly covered with setae; coxa with a macroseta near dorsomedial of anterior surface and 5–6 long setae at apical margin; femur with longer setae near anterior inner margin; tibia with apical setae stronger, 2 spurs present at apex. Mid leg (Fig. 15) uniformly covered with setae except coxa; coxa with several long setae around anterior margin; tibia with long macroseta at middle on dorsal surface, 3 spurs present at apex. Hind leg (Fig. 16) uniformly covered with setae, coxa medially nude; coxa with 3 longer setae along cavity; trochanter and femur with a macroseta near middle of anterior surface; tibia with one longer and shorter macroseta on anterior surface, 3 apical spurs present.

Tergites and paratergites III–VIII (Figs 1, 17) sparsely covered with setae. Macrochaetotaxy of abdominal tergites III–VIII = 0-0-2-2-0-2. Sternite VIII (Fig. 18) sparsely covered with setae; 3 pairs of macrosetae present, 2 pairs at mediolateral margin and one near apex. Tergite IX (Fig. 19) with pores around anterior margin, several setae near middle of disc, 2 pairs of macrosetae at mediolateral margin and apex present. Tergite X (Fig. 19) covered in setae, 4 longer setae at apex, 2 pairs of macrosetae present at mediolateral margin and apex.

*Male*. Median lobe of aedeagus (Figs 20, 21) copulatory piece flagellate, suspensoria associated with lateral base of copulatory piece. Paramere (Fig. 22) apical lobe with pores at base.

*Female*. Spermatheca (Fig. 23) cuticle at apex with wrinkle-like sculpture.

#### Etymology.

The specific epithet is derived from its type locality of Bolivia Latinized. The gender is masculine.

#### Distribution.

Cochabamba, Bolivia.

#### Ecology.

All known specimens of *Neotermitosocius bolivianus* were collected with a passive sample method (flight intercept trap) and nothing is known about its biology. It is hypothesized to be a termite symbiont based on phylogenetic relationships but the host is unknown.

### Coptotermocola

Kanao, Eldredge & Maruyama gen. n.

urn:lsid:zoobank.org:act:01A98C55-BA86-4064-A554-1EC1DA6DC93A

http://species-id.net/wiki/Coptotermocola

#### Type species:

*Coptotermocola clavicornis* sp. n.

#### Diagnosis.

This monotypic genus is distinguishable from all other Termitohospitini most distinctly by the strongly carinate mesocoxal process (Figs 27, 38), and additionally by the compact antennae (Fig. 29) and short maxillary palpi (Fig. 33).

#### Description.

**Figures 24–27.**
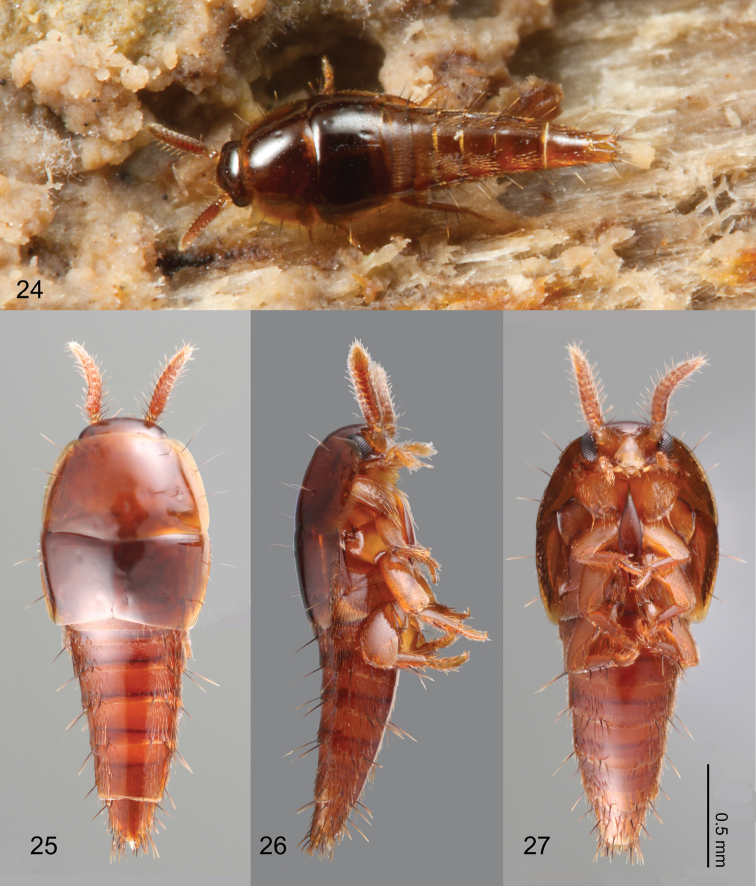
Habitus of *Coptotermocola clavicornis*. **24** beetle photographed *in situ* **25** dorsal view **26** lateral view **27** ventral view.

Body form (Figs 24–27) limuloid. Pronotum strongly convex, dorsally obscuring most of head; abdomen posteriorly tapered.

**Figure 28–29. F7:**
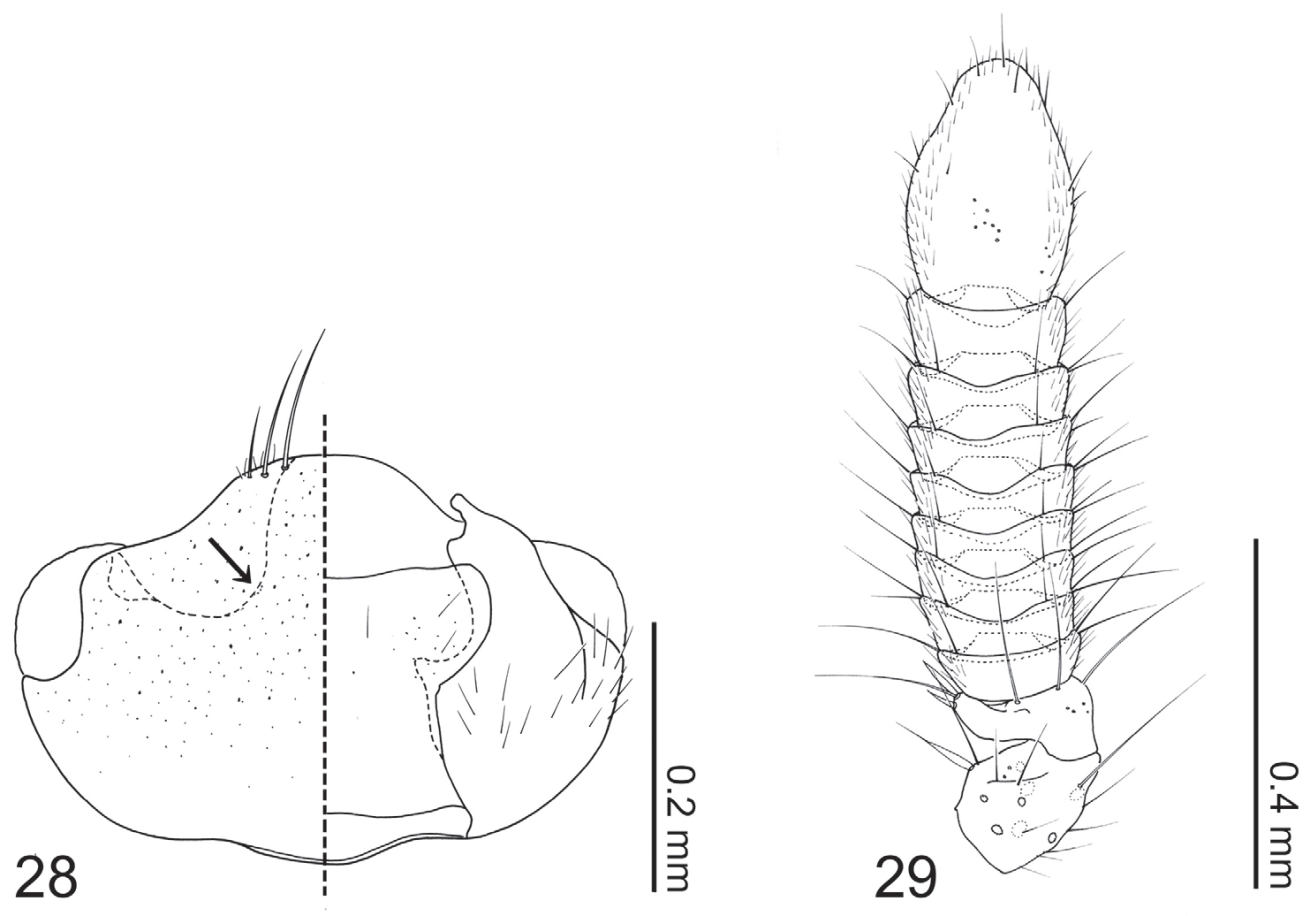
*Coptotermocola clavicornis*. **28** head capsule left side = dorsal view, right side = ventral view **29** antenna.

**Figures 30–35. F8:**
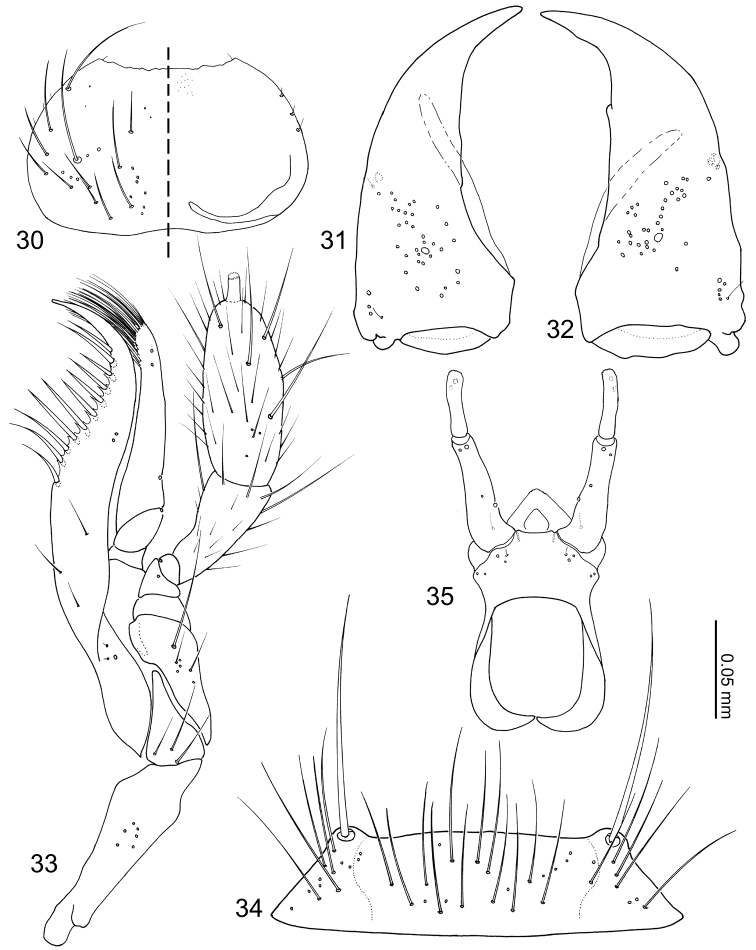
*Coptotermocola clavicornis*, mouthparts. **30** labrum, left side = labrum, right side = epipharynx **31** left mandible, dorsal view **32** right mandible, dorsal view **33** maxilla, ventral view **34** mentum, ventral view **35** labium, ventral view.

Head capsule (Fig. 28) transverse, widest behind eyes. Antennal fossae deep, as large as eyes and dorsally obscured by vertex. Eyes large and produced anterolaterally. Antennae (Fig. 29) compact with 11 articles; antennomere I longer than II–X and partially hidden within antennal fossae; antennomere II dilated at apex to receive antennomere III; antennomeres III–X strongly transverse and more than twice as wide as long, length increasing distally; antennomere XI fusiform; pedicles of antenomeres IV–XI obscured by apex of preceding article. Labrum (Fig. 30) transverse and semicircular, anterior margin broadly concave; disc basomedially sparsely covered with pores; epipharynx (Fig. 30) glabrous. Mandibles (Figs 31–32) asymmetrical, dorsally covered with numerous pores around middle. Right mandible (Fig. 32) with a small subapical tooth; anterior one fifth strongly curved adorally. Left mandible (Fig. 31) without a subapical tooth. Maxillary (Fig. 33) lacinia elongate and strongly recurved adorally, adoral margin with 11 setae; apical two setae without basal cuticular processes (spinose scale of Ahn and Ashe 2004), third apical seta with a proximal basal cuticular process, central five setae with paired basal cuticular processes, basal three setae without basal cuticular processes; galea tapered apically, apex densely furnished with long trichae; palpus with 4 articles; article I triangular; article II trapezoidal and dilated toward apex; article III oviform, more than three times longer than wide and sparsely covered with long setae; article IV slightly narrowed toward apex with inconspicuous pseudosegment, pseudopores present apicomedially. Mentum (Fig. 34) trapezoidal, disc covered with long setae and several pores near middle and lateral areas; anterolateral corners slightly produced and with a pair of long macrosetae. Labial (Fig. 35) palpus with 3 articles, first article a fusion of I + II; article I + II with a mediobasal seta on ventral surface (probably homologous to seta c of Sawada [1972]), twin and medial pores present; article III almost half as long as article I + II with 2 pores at apex; ligula triangular with medial subtriangular area sclerotized; prementum wider than long, with 2 pairs of both real and pseudopores, and a pair of setal pores present; apodemes broad and longer than disc, dilated posteriorly with apices recurved internally and almost touching.

**Figures 36–42. F9:**
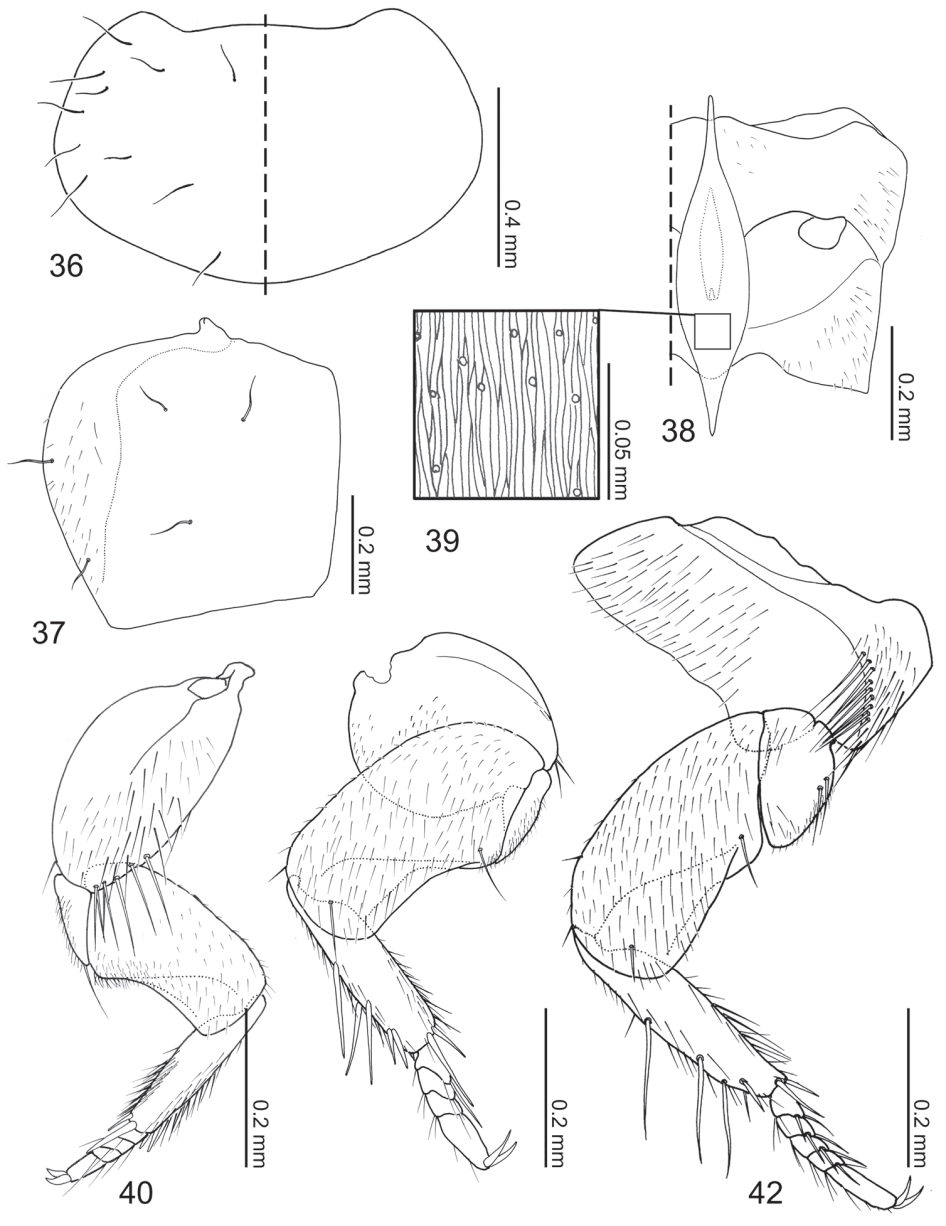
*Coptotermocola clavicornis*, thorax and legs. **36** pronotum, left side with and right side without setae **37** elytron, left **38** meso- and metaventrites, anatomical left side with setae **39** detail of mesosternal process surface sculpture **40** fore leg, posterior surface **41** mid leg, posterior surface **42** hind leg, posterior surface.

Pronotum (Fig. 36) transverse, widest at posterolateral angles; anterior margin concave with anterolateral angles slightly produced anteriorly; posterior margin rounded and slightly produced medially; marginal cuticle thin and somewhat translucent (Fig. 26). Prosternum reduced in length. Elytra (Fig. 37) subquadrate, slightly wider than long. Wings fully developed. Mesoventrite (Fig. 38) slightly shorter than metaventrite; mesoventral process produced as an extremely large structure widest medially and tapered at ends, microsculpture composed of longitudinal wavy lines and pores (Fig. 39), structure ventrally partially obscuring basal podomeres; mesocoxal cavity marginal bead complete, narrowly separated. Legs (Figs 40–42) stout and laterally flattened; femora subrectangular; apical tarsomeres longest, tarsal formula 4-4-5. Fore leg (Fig. 40) with coxa approximately as long as femur; trochanter subtriangular; tibia thin. Mid leg (Fig. 41) with coxa globular; trochanter very thin; tibia thin. Hind leg (Fig. 42) subrectangular and almost as long as trochanter and femur combined; trochanter globular-triangular; femur slightly dilated apically; tibia thin.

**Figures 43–49.**
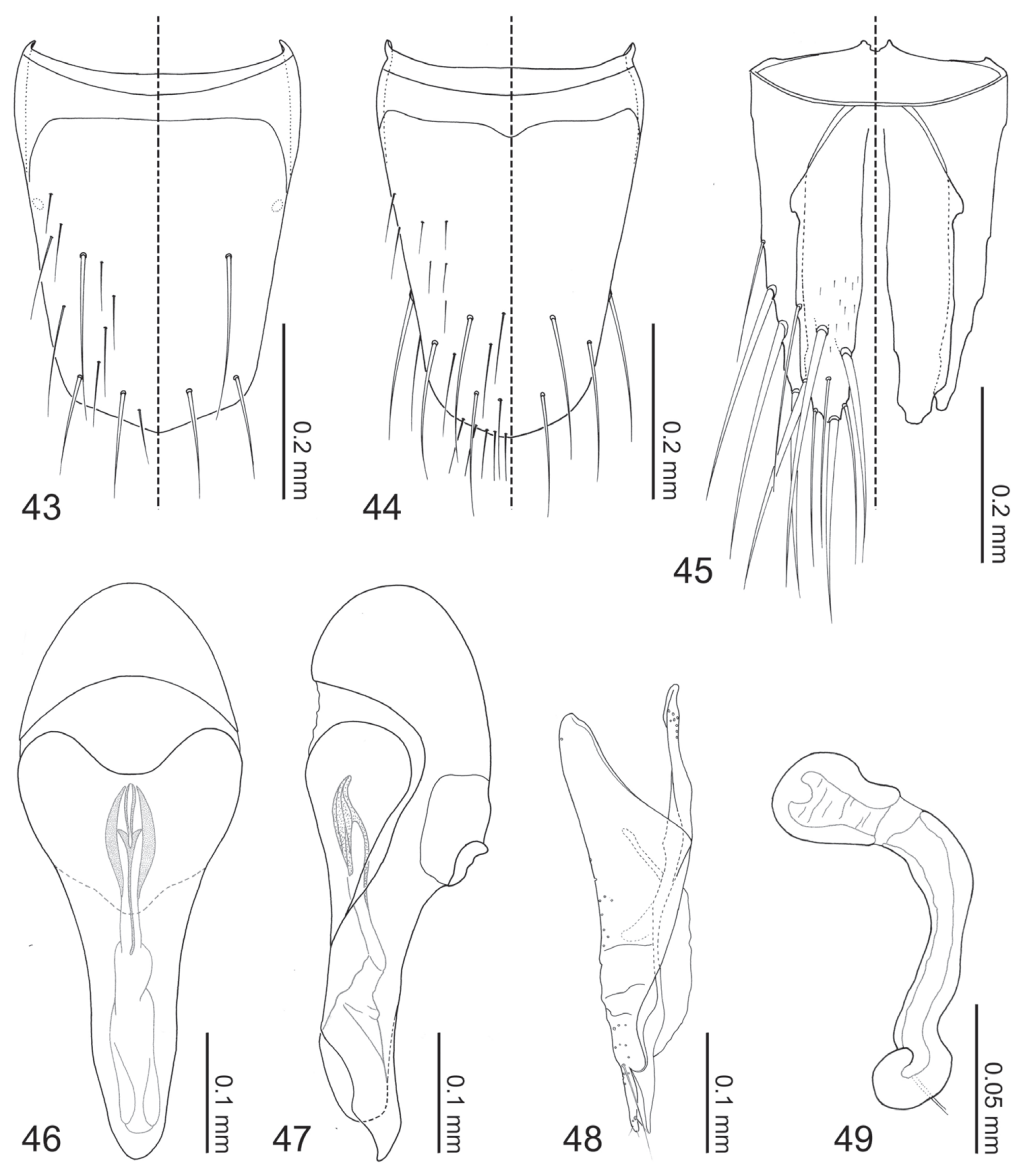
*Coptotermocola clavicornis*, abdominal sclerites and genitalia. **43** tergite VIII **44** sternite VIII **45** tergites IX–X **46** median lobe of aedeagus, aparameral view **47** median lobe of aedeagus, lateral view **48** paramere, external view **49** spermatheca.

Abdomen (Figs 24–27) tapered posteriorly. Segment I represented by only tergite I fused to the metanotum. Segment II represented by only by tergite II. Segments III–VII with 1 tergite, 1 sternite, and 2 pairs of paratergites. Tergite VIII (Fig. 43) with a blunt apicomedial point. Sternite VIII (Fig. 44) with posterior margin rounded. Tergite IX (Fig. 45) fully subdivided dorsally by tergite X. Tergite X (Fig. 45) fully divided medially to base, only connected each other and tergite IX on anterior margin.

Median lobe of aedeagus (Figs 46–47) with basal capsule bulbous. Paramere (Fig. 48) with a structure appearing homologous to Seevers’ (1978) medial phragma produced apically. Spermatheca (Fig. 49) apically slightly bulbous.

#### Etymology.

The generic name is derived from a combination of the generic name of the host termite, *Coptotermes* Wasmann, 1896 and the Latin noun *cola* meaning “dweller”. The gender is feminine.

### Coptotermocola clavicornis

Kanao, Eldredge & Maruyama sp. n.

urn:lsid:zoobank.org:act:FEF68FC3-CB00-4B41-95C8-A6DA28E8FFA7

http://species-id.net/wiki/Coptotermocola_clavicornis

Figures 24 [Fig F7] [Fig F8] [Fig F9] –49

#### Type material.

*Holotype*:♂, “MALAYSIA: Selangor,/Ulu Gombak, 03°19'479"N; 101°45'170"E,/ca. 240 m alt., X July 2011,/T. Kanao leg. KT-261”. Abdominal segments VIII–X dissected off.

*Paratypes*:6??, MALAYSIA: same data as the holotype, one specimen is preserved in 99.5% EtOH; ♀, same locality data as the holotype, differing data reads “XXI May 2010,/ T. Kanao leg. KT-33”, fully disarticulated; 3??, same locality data as the holotype, differing data reads “XXIX May 2012,/ T. Kanao leg. KT-312”, one specimen is preserved in 99.5% EtOH.

All type specimens are deposited in the Kyushu University Museum.

#### Diagnosis.

This species is diagnosable based on the generic diagnosis above.

#### Description.

Body (Figs 24–27) approximately 2 mm in length (1.71–2.16 mm, N = 4) almost uniformly reddish brown, but head slightly darker. Dorsal surface of head (Fig. 28) glabrous, sparsely covered with pores, with 3 pairs of long setae at anterior margin of clypeus; ventral surface (Fig. 28) with several setae behind eyes. Antennomere (Fig. 29) I sparsely covered with pseudopores and several macrosetae; antennomere II with 6–7 long macrosetae, 2 of them stronger and several pores present; antennomeres III–X sparsely covered with setae and 3–4 macrosetae present; antennomere XI sparsely covered with setae, with several macrosetae on dorsal and ventral surface near apex, pores present centrally on lateral surface. Labral (Fig. 30) surface with 14–16 setae, anterolateral marginal and near-middle pairs conspicuously stronger. Epipharynx (Fig. 30) with a pair of setulae present on anterolateral corner and three pairs of lateral marginal setulae. Mandibles (Figs 31–32) with seta present at aboral basolateral margin. Maxillary (Fig. 33) lacinia mesally with two pores and basally with 3 setae present; galea with 2 pores apically; maxillary palpal article I with a medial pore, article II sparsely covered with setae and longer setae present on apical margin, article III sparsely covered with longer and shorter setae.

Pronotum (Fig. 36) transverse (pronotum length = 0.55–0.62 mm, pronotum width = 0.91–1.02 mm, N = 6) with 11 pairs of macrosetae. Elytra (Fig. 37) subquadrate (elytra length = 0.50–0.60 mm, elytra width = 0.51–0.63, N = 6), disc laterally sparsely setose, 2 lateral and 3 discal pairs of macrosetae present. Mesoventrite (Fig. 38) with central and lateral setose areas. Metaventrite (Fig. 38) with posterolateral setose area. Fore leg (Fig. 40) with coxa sparsely setose and 5 long macrosetae present at apical margin; trochanter and femur sparsely covered with setae; tibia covered with setae, density increasing apically, 5 apical spurs present; tarsus with few setae. Mid leg (Fig. 41) with coxa sparsely setose, 2 macrosetae at apex; trochanter sparsely covered with setae; femur overall setose, macroseta present venterobasally; tibia covered with setae, density increasing apically, with 7 strong setae present dorsally and apically with basal three dorsal setae longest; tarsomeres with 3–4 setae at apical margin. Hind leg (Fig. 42) with coxa mostly setose and with approximately 10 macrosetae along femoral cavity margin; trochanter partially setose and with 2 macrosetae along ventrolateral margin; femur overall setose, one macroseta near base and three macrosetae apically present; tibia sparsely covered with setae, with 7 strong setae present dorsally and apically, basal three dorsal setae longest; tarsomeres with 3–4 micro- and 2 macrosetae present at apical margin.

Tergites III–VIII (Fig. 25, 43) laterally setose and medially glabrous. Macrochaetotaxy of abdominal tergites III–VIII = 2-4-4-4-4-4; paratergites setose. Tergite VIII (Fig. 43) with a pair of discal and two pairs of apical macroseta present. Sternite VIII (Fig. 44) sparsely setose and with one discal and three marginal macrosetae. Tergite IX (Fig. 45) with 3 pairs of macrosetae at apex and lateral margin; tergite X (Fig. 45) disc sparsely covered with minute setae and 4 pair of macrosetae near apex.

*Male*. Median lobe of aedeagus (Figs 46–47) copulatory piece flagellate, suspensoria associated with lateral base of copulatory piece. Paramere (Fig. 48) condylite with pores basally; apical lobe with 4 setae present.

*Female*. Spermatheca (Fig. 49) apical bulb surface with transverse wrinkle-like sculpture; stalk basal to membranous area three times as long as apical bulb.

#### Etymology.

The specific epithet is derived from a combination of the Latin noun *clava* meaning “club” and Latin adjective *cornis* meaning “to be horned”, in reference to the diagnostic robust antennae of the species. The gender is feminine.

#### Distribution.

Known only from the type locality Ulu Gombak, Selangor, Malaysia.

#### Host species.

All specimens were collected from the nest of *Coptoptermes gestroi* (Wasmann, 1896). Ahmad (1965), Kirton and Brown (2003) and Tho (1992) was consulted for host identification.

#### Ecology.

Specimens acquired during the KT261 collecting event were collected from the galleries of the host termites within a rotting log. The galleries were large and arranged in a complex manner. Another specimen (KT33) was collected from a trail of the hosts that occupied the exterior of a large log. KT312 specimens were collected from a rotting log occupied by the host termites. All *Coptotermes* colonies that yielded *Coptotermes clavicornis* were located near rivers where the habitat in general was comparatively more moist compared to its surroundings.

All *Coptotermes clavicornis* specimens moved faster than their host termites. They did not avoid contact with hosts but instead recurved their abdomens over their bodies when they came into contact. The inquilines wedged themselves under their hosts on several occasions, but the host termites regaurdless never attacked the beetles.

## Supplementary Material

XML Treatment for Termitohospitini

XML Treatment for Neotermitosocius

XML Treatment for Neotermitosocius bolivianus

XML Treatment for Coptotermocola

XML Treatment for Coptotermocola clavicornis
